# Supplementary figures and images for: IQGAP3 in clear cell renal cell carcinoma contributes to drug resistance and genome stability
Source: PeerJ. 2022 Oct 18;10:e14201. doi: 10.7717/peerj.14201 (PMC9586079; doi:10.7717/peerj.14201)

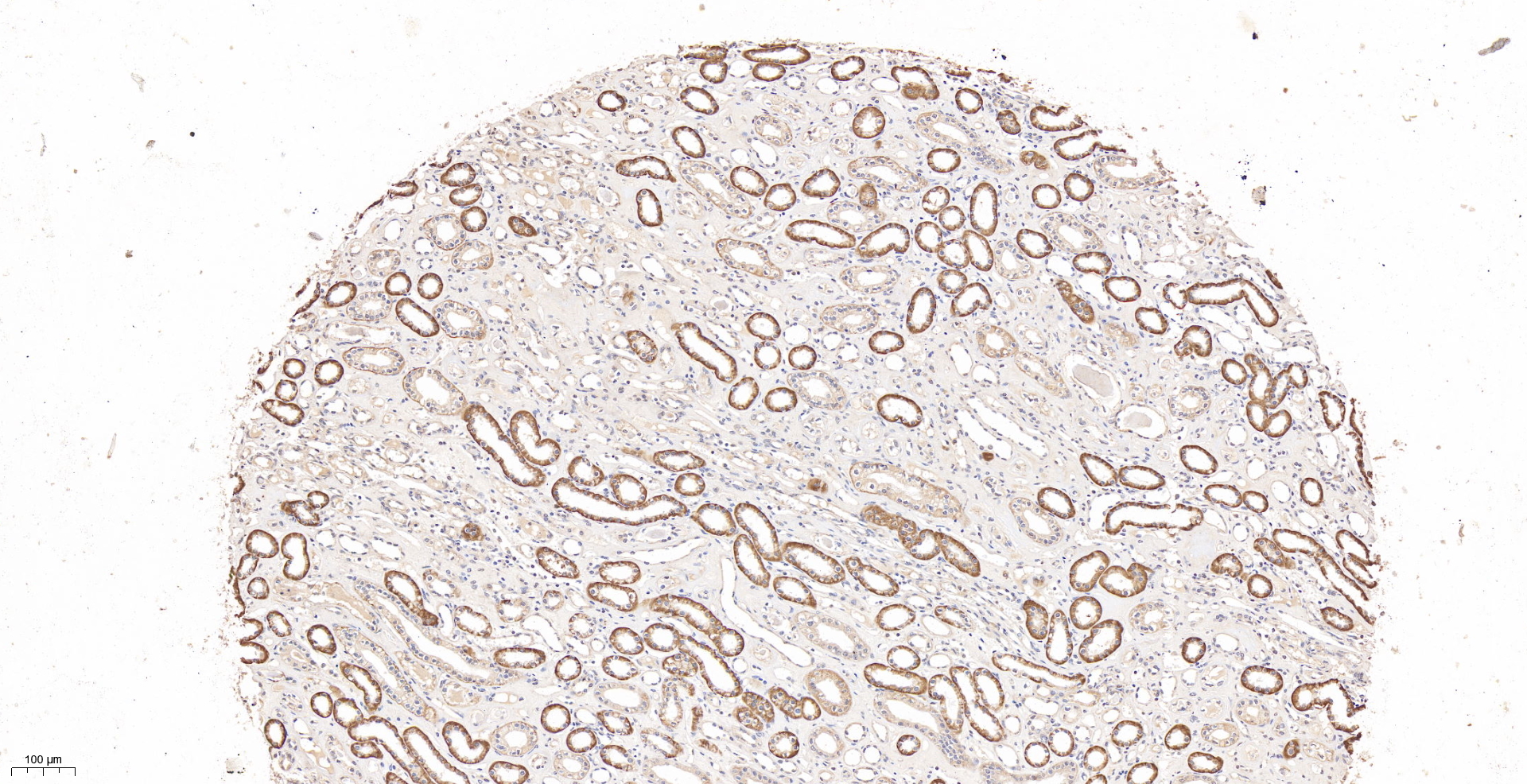

Supplement: Supplemental Information 1 [file peerj-10-14201-s001.zip › Supplemental file Raw data-0824/Supplemental file-Raw data for Figure 1H-Normal-1.jpg]

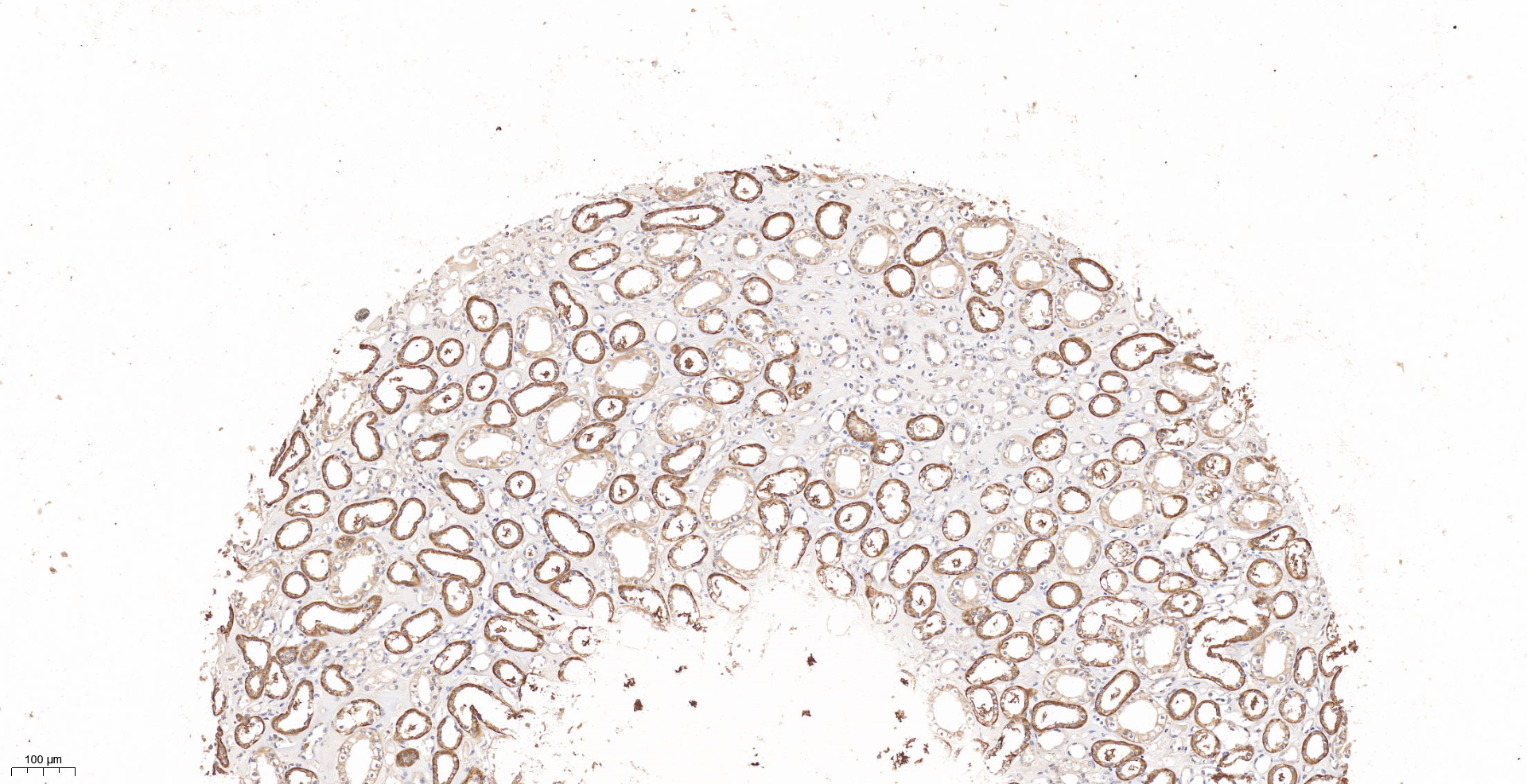

Supplement: Supplemental Information 1 [file peerj-10-14201-s001.zip › Supplemental file Raw data-0824/Supplemental file-Raw data for Figure 1H-Normal-2.jpg]

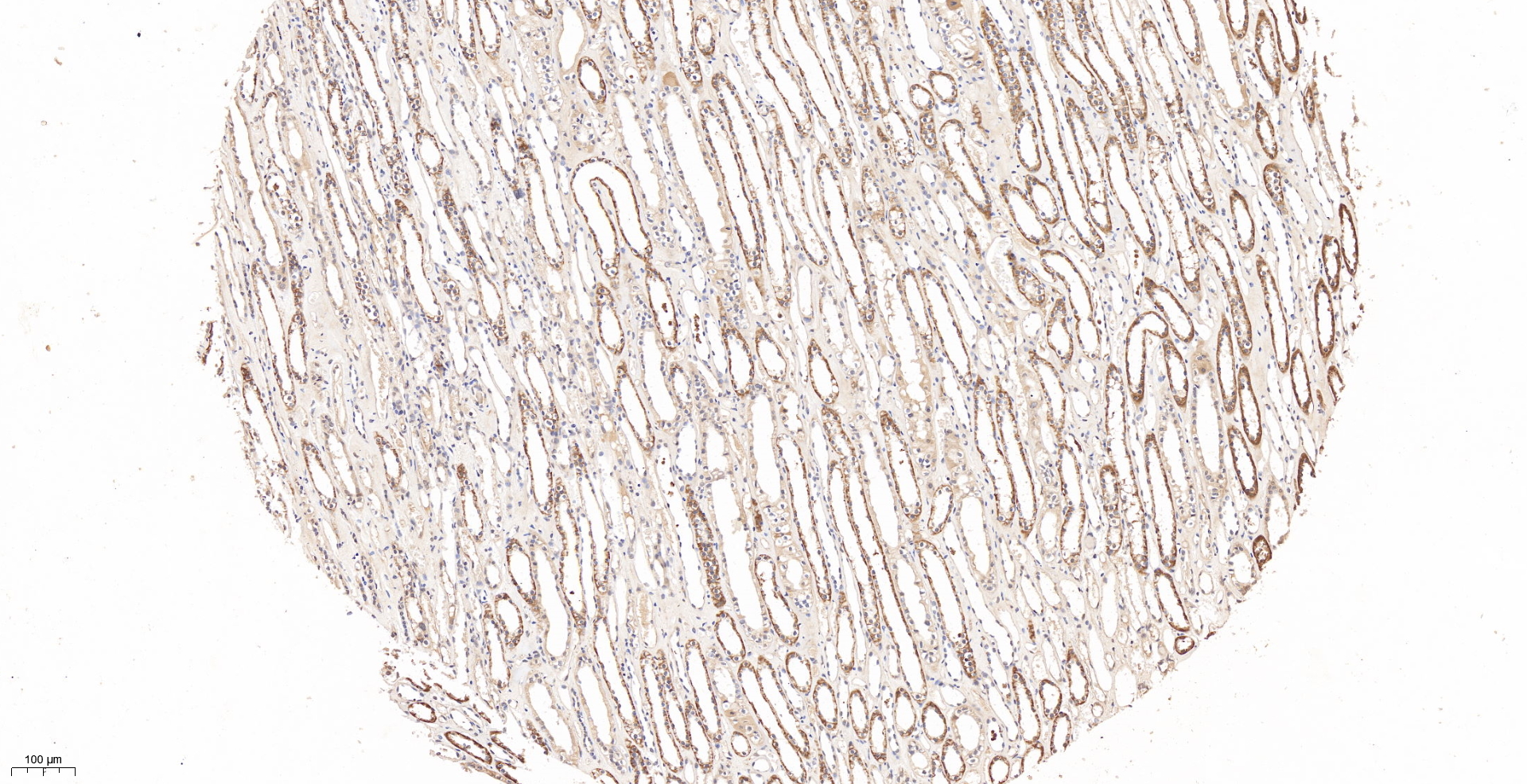

Supplement: Supplemental Information 1 [file peerj-10-14201-s001.zip › Supplemental file Raw data-0824/Supplemental file-Raw data for Figure 1H-Normal-3.jpg]

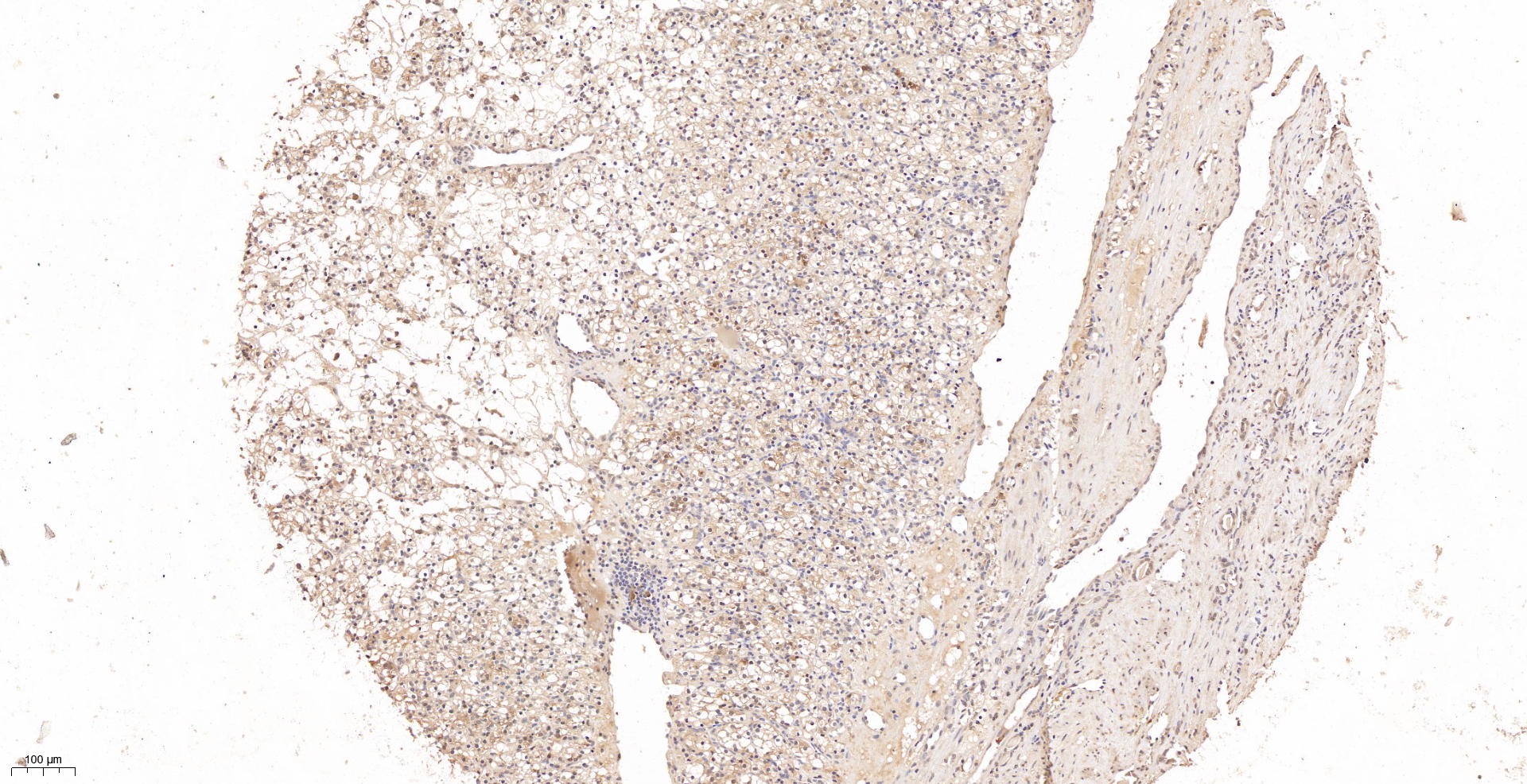

Supplement: Supplemental Information 1 [file peerj-10-14201-s001.zip › Supplemental file Raw data-0824/Supplemental file-Raw data for Figure 1H-Tumor-1.jpg]

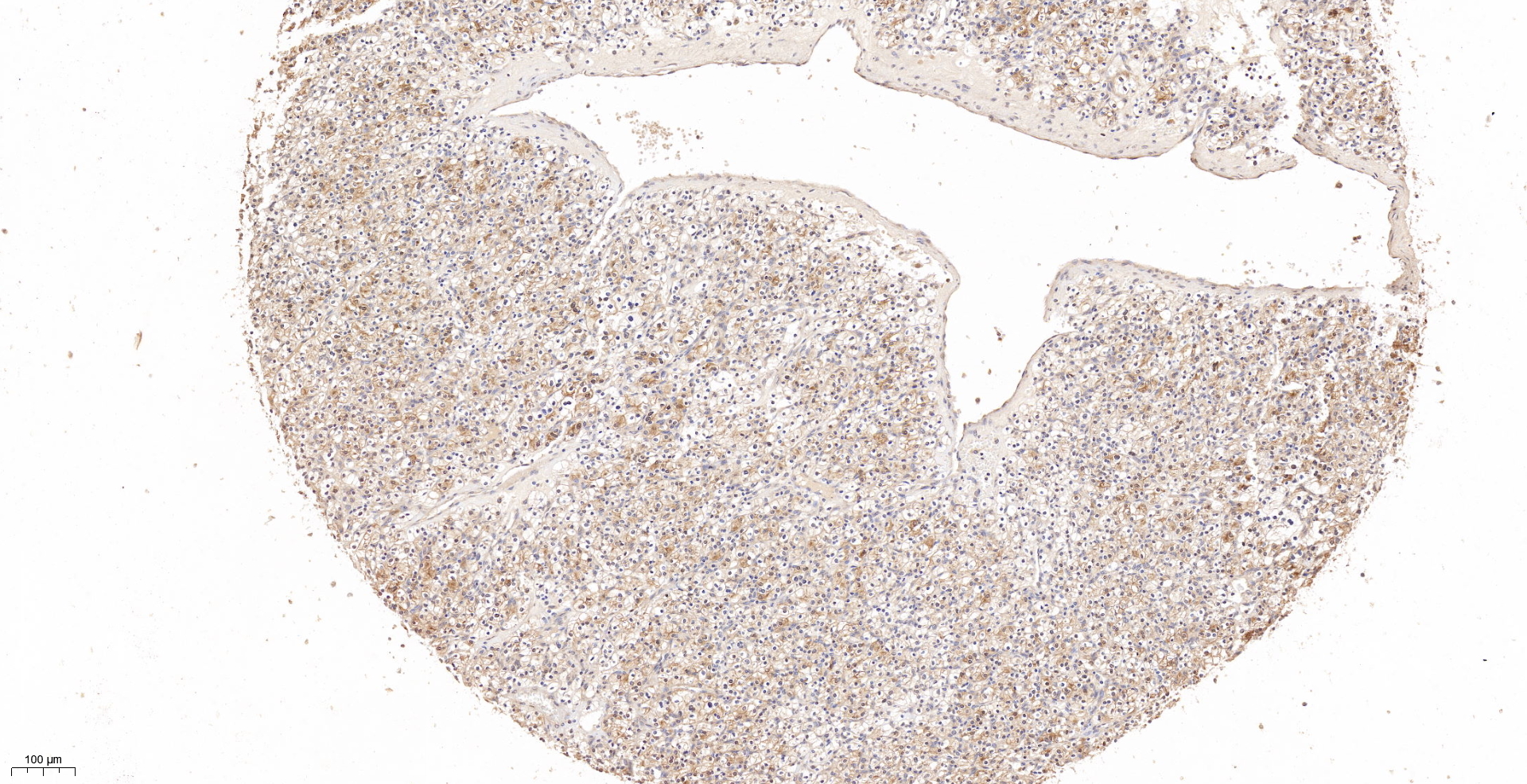

Supplement: Supplemental Information 1 [file peerj-10-14201-s001.zip › Supplemental file Raw data-0824/Supplemental file-Raw data for Figure 1H-Tumor-2.jpg]

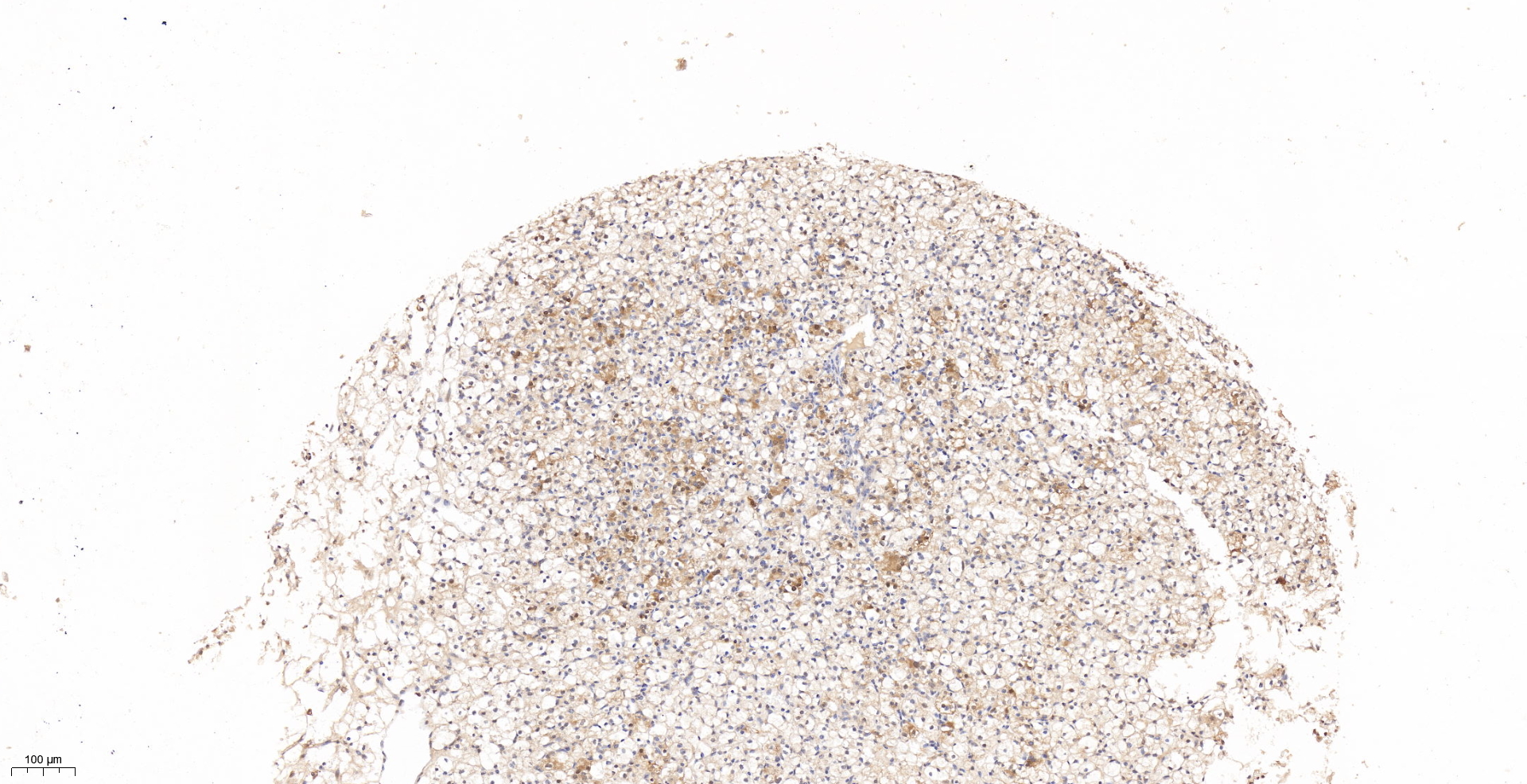

Supplement: Supplemental Information 1 [file peerj-10-14201-s001.zip › Supplemental file Raw data-0824/Supplemental file-Raw data for Figure 1H-Tumor-3.jpg]

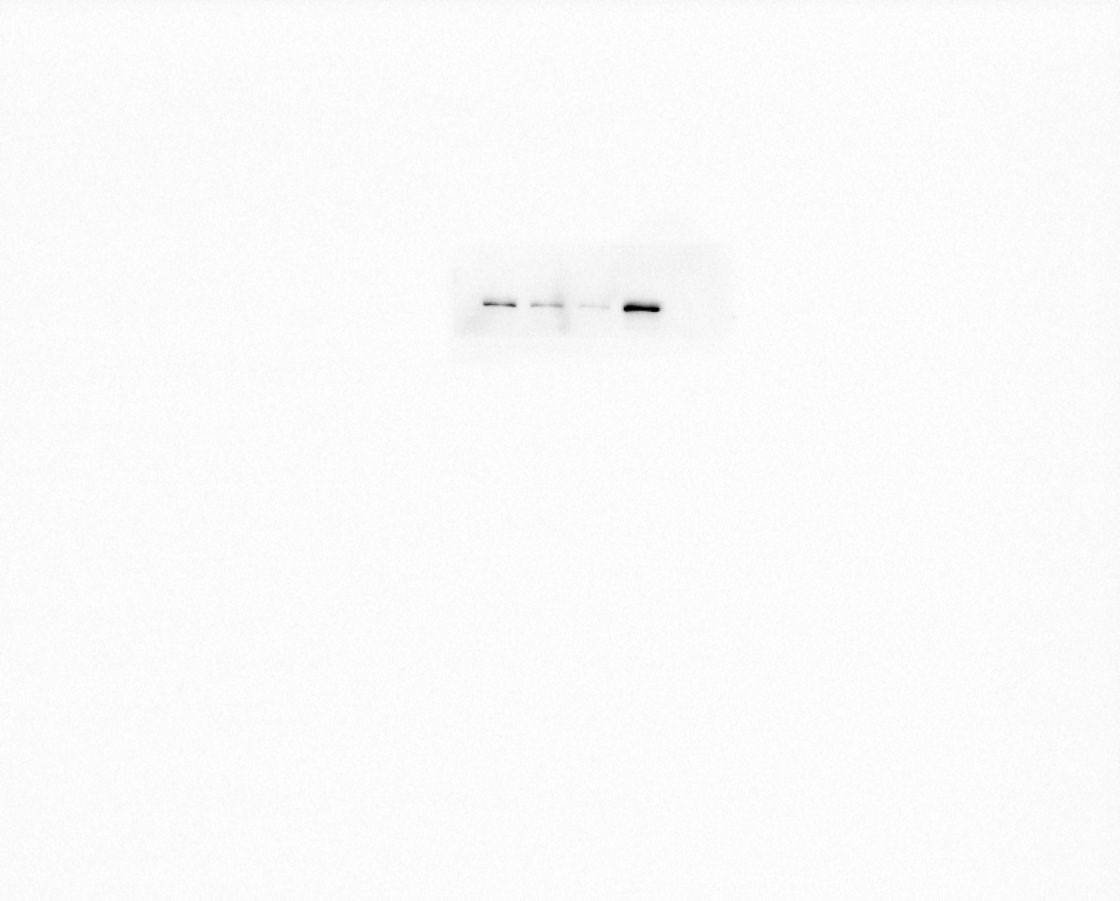

Supplement: Supplemental Information 1 [file peerj-10-14201-s001.zip › Supplemental file Raw data-0824/Supplemental file-Raw data for Figure 4A-1.jpg]

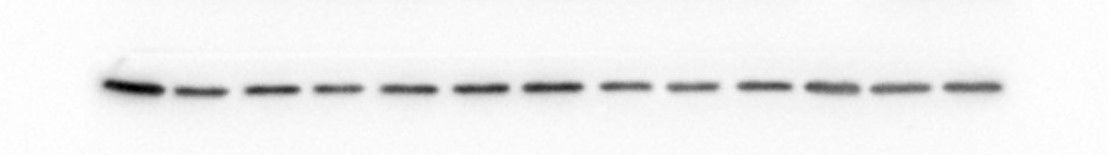

Supplement: Supplemental Information 1 [file peerj-10-14201-s001.zip › Supplemental file Raw data-0824/Supplemental file-Raw data for Figure 4A-2.jpg]

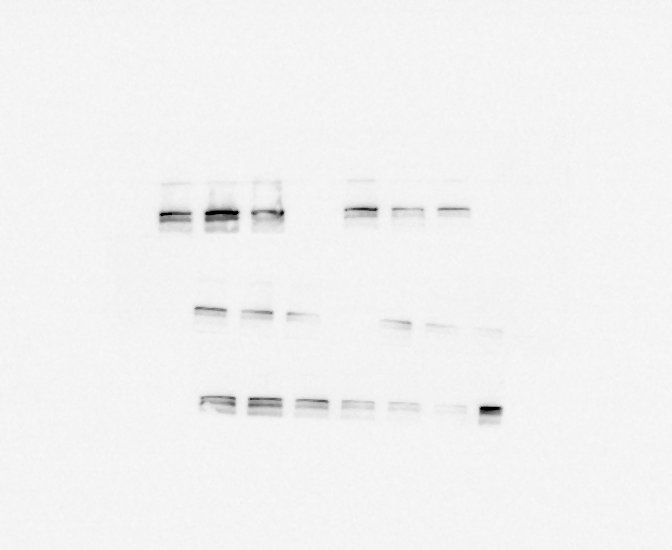

Supplement: Supplemental Information 1 [file peerj-10-14201-s001.zip › Supplemental file Raw data-0824/Supplemental file-Raw data for Figure 4A-4.jpg]

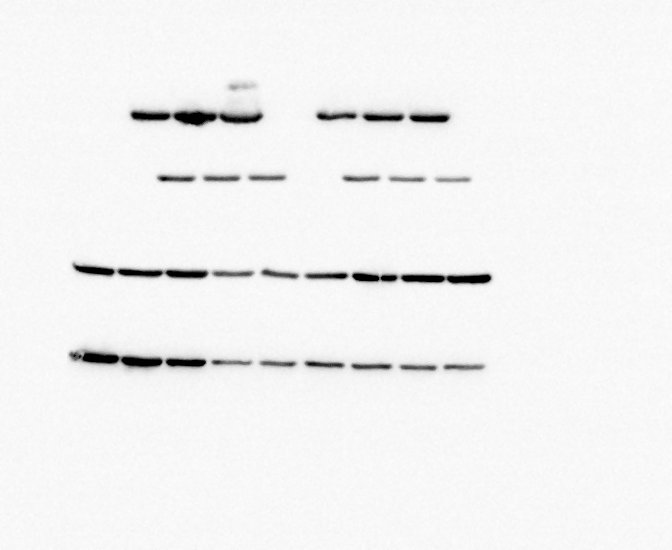

Supplement: Supplemental Information 1 [file peerj-10-14201-s001.zip › Supplemental file Raw data-0824/Supplemental file-Raw data for Figure 4A-5.jpg]

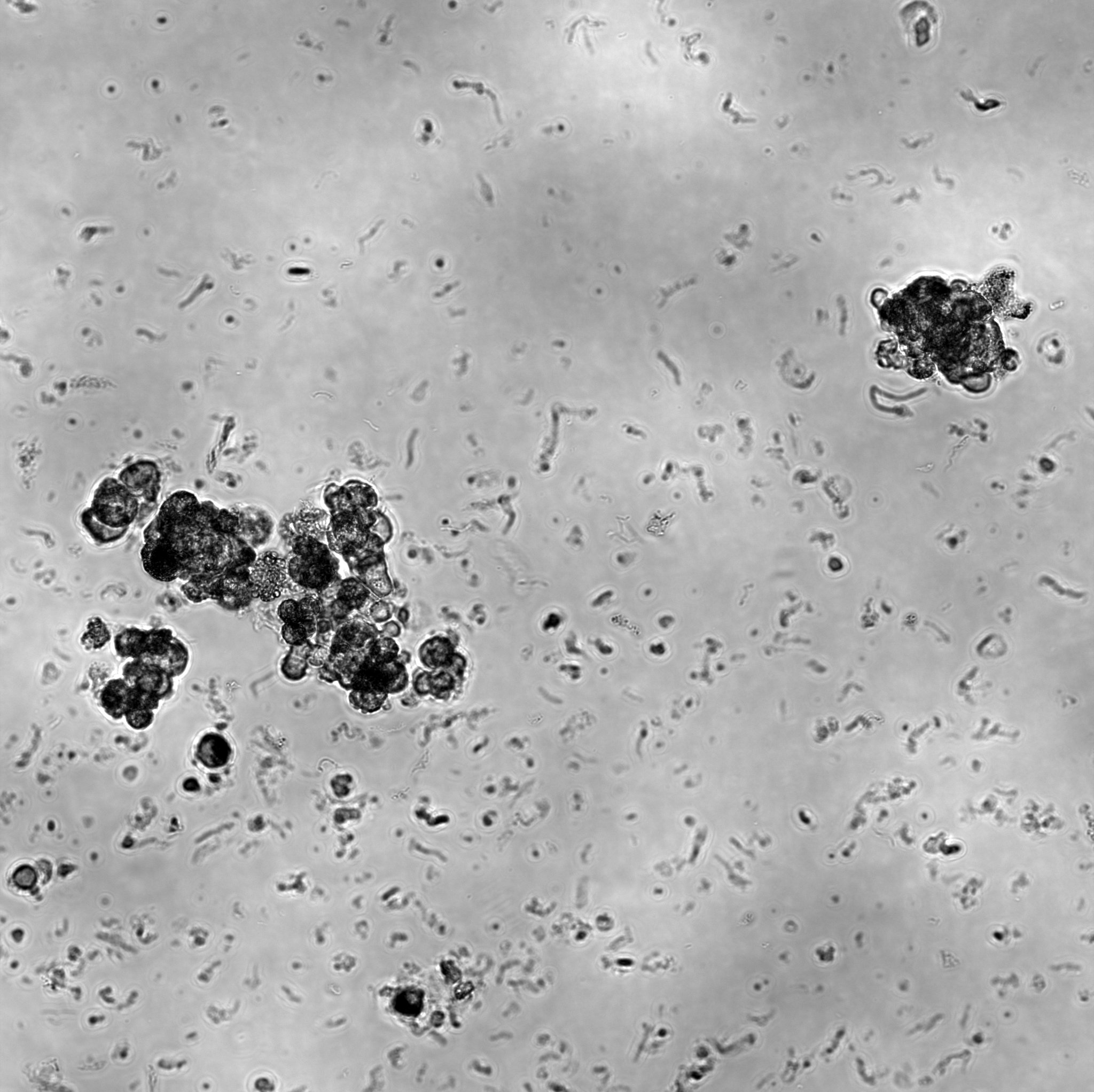

Supplement: Supplemental Information 1 [file peerj-10-14201-s001.zip › Supplemental file Raw data-0824/Supplemental file-Raw data for Figure 4C-1.jpg]

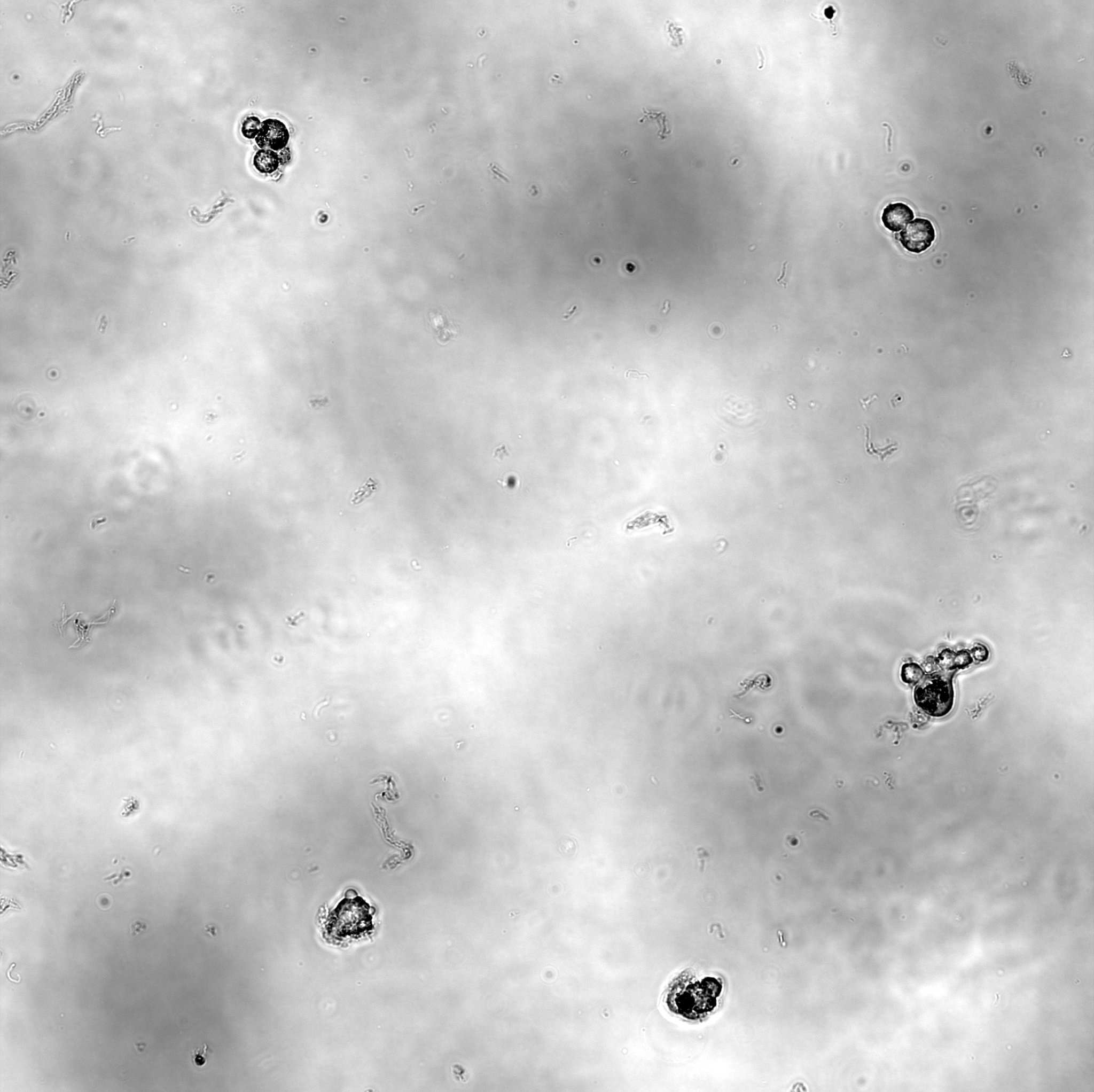

Supplement: Supplemental Information 1 [file peerj-10-14201-s001.zip › Supplemental file Raw data-0824/Supplemental file-Raw data for Figure 4C-2.jpg]

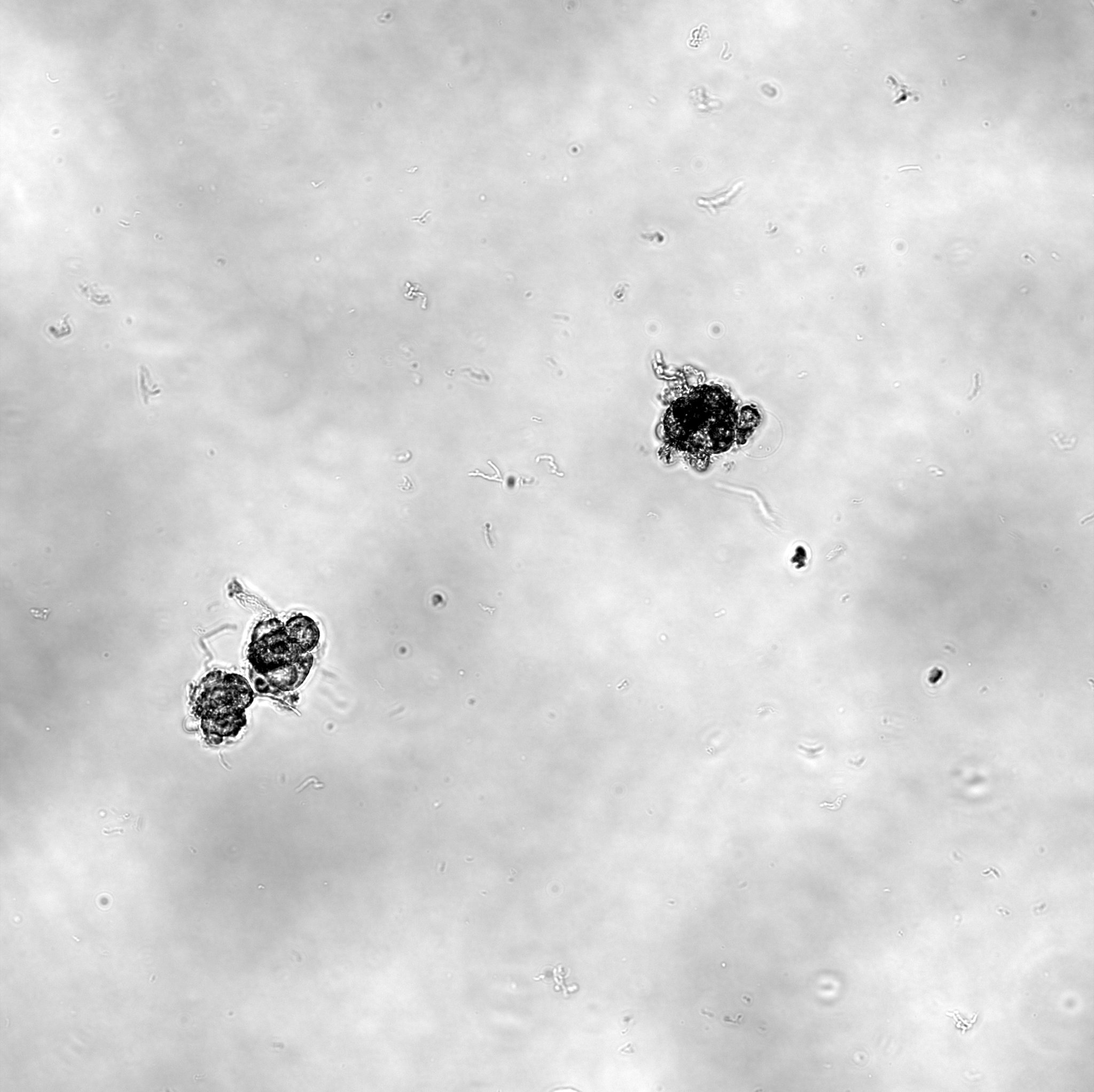

Supplement: Supplemental Information 1 [file peerj-10-14201-s001.zip › Supplemental file Raw data-0824/Supplemental file-Raw data for Figure 4C-3.jpg]

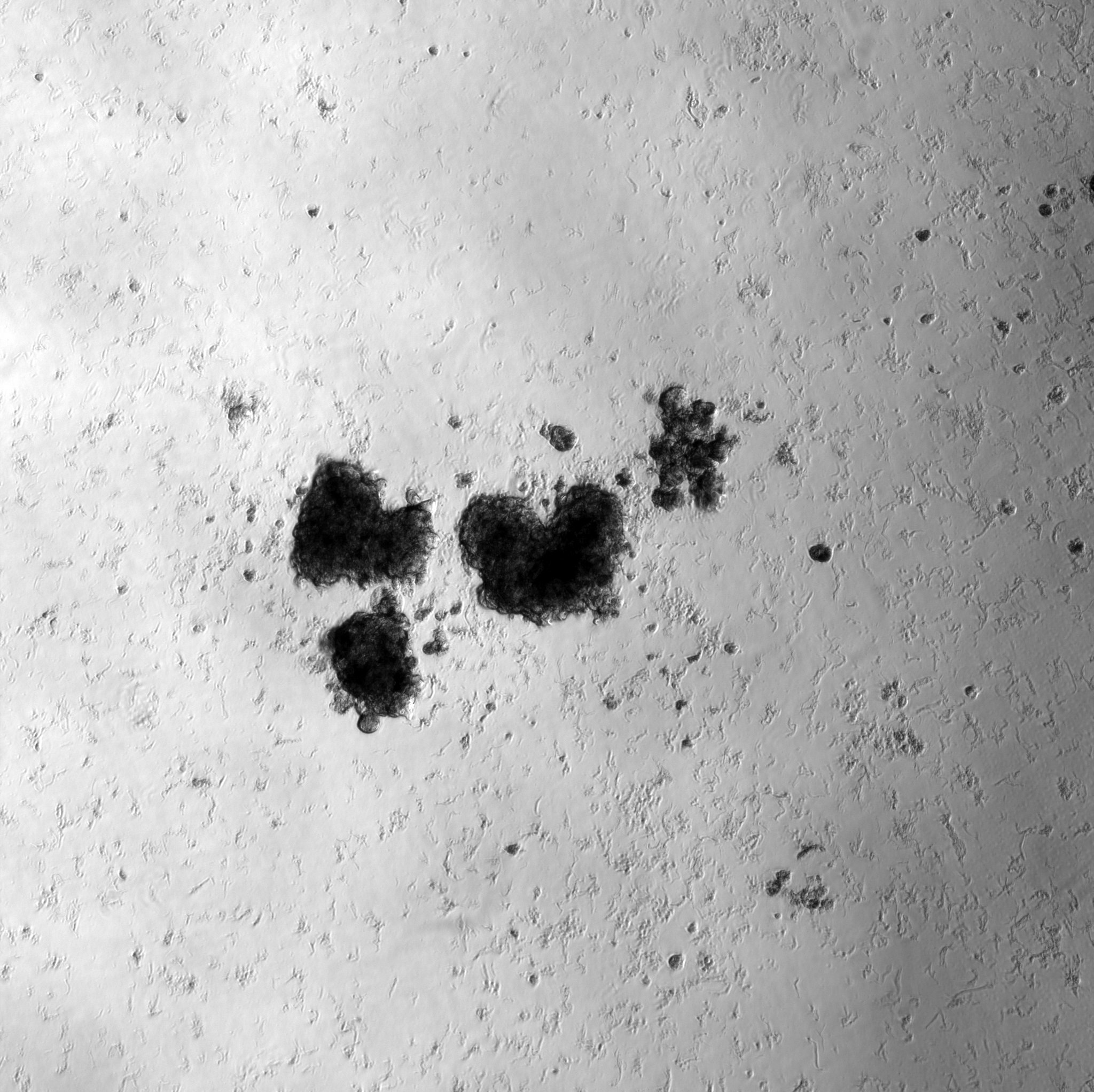

Supplement: Supplemental Information 1 [file peerj-10-14201-s001.zip › Supplemental file Raw data-0824/Supplemental file-Raw data for Figure 4C-4.jpg]

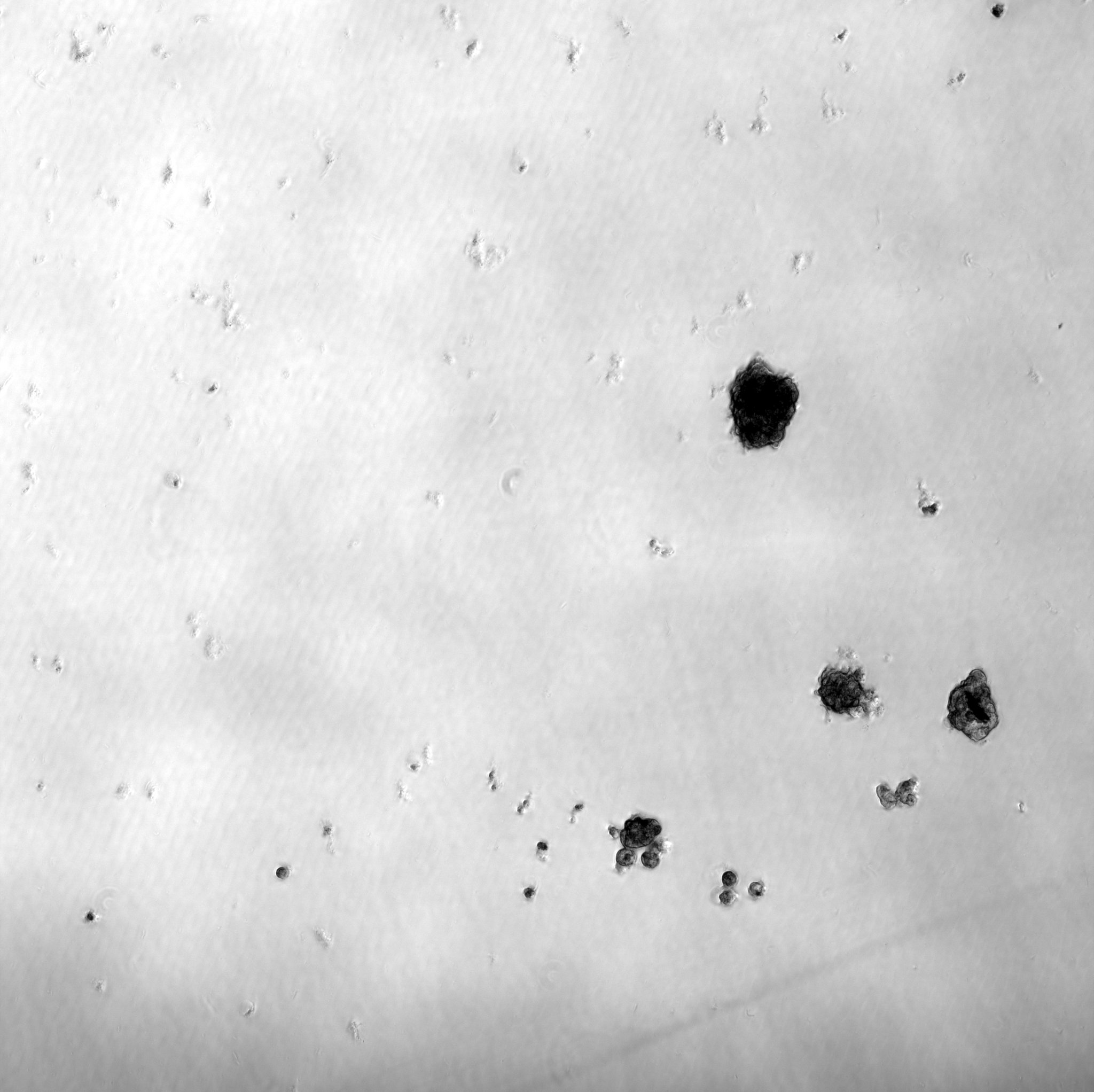

Supplement: Supplemental Information 1 [file peerj-10-14201-s001.zip › Supplemental file Raw data-0824/Supplemental file-Raw data for Figure 4C-5.jpg]

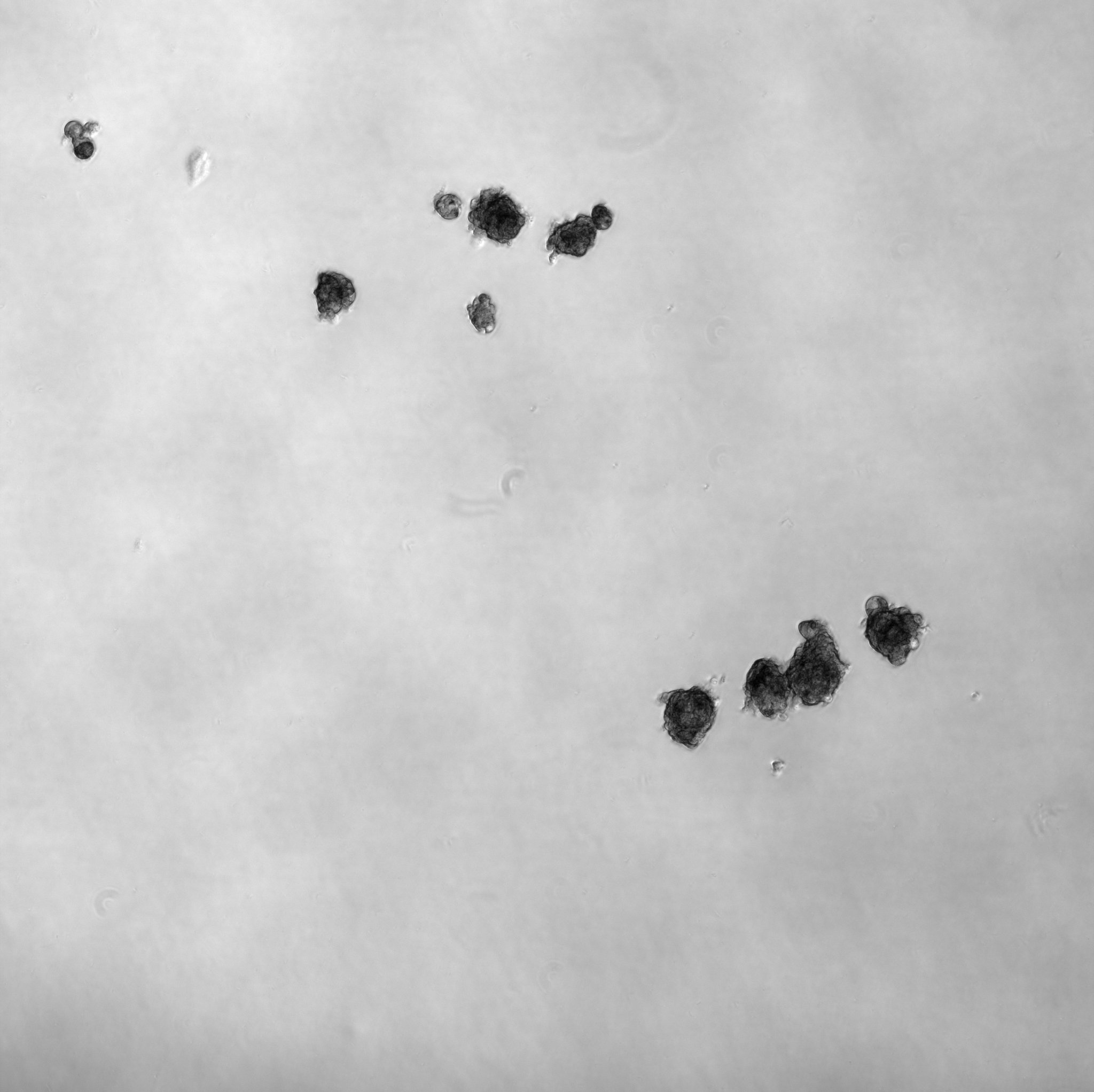

Supplement: Supplemental Information 1 [file peerj-10-14201-s001.zip › Supplemental file Raw data-0824/Supplemental file-Raw data for Figure 4C-6.jpg]

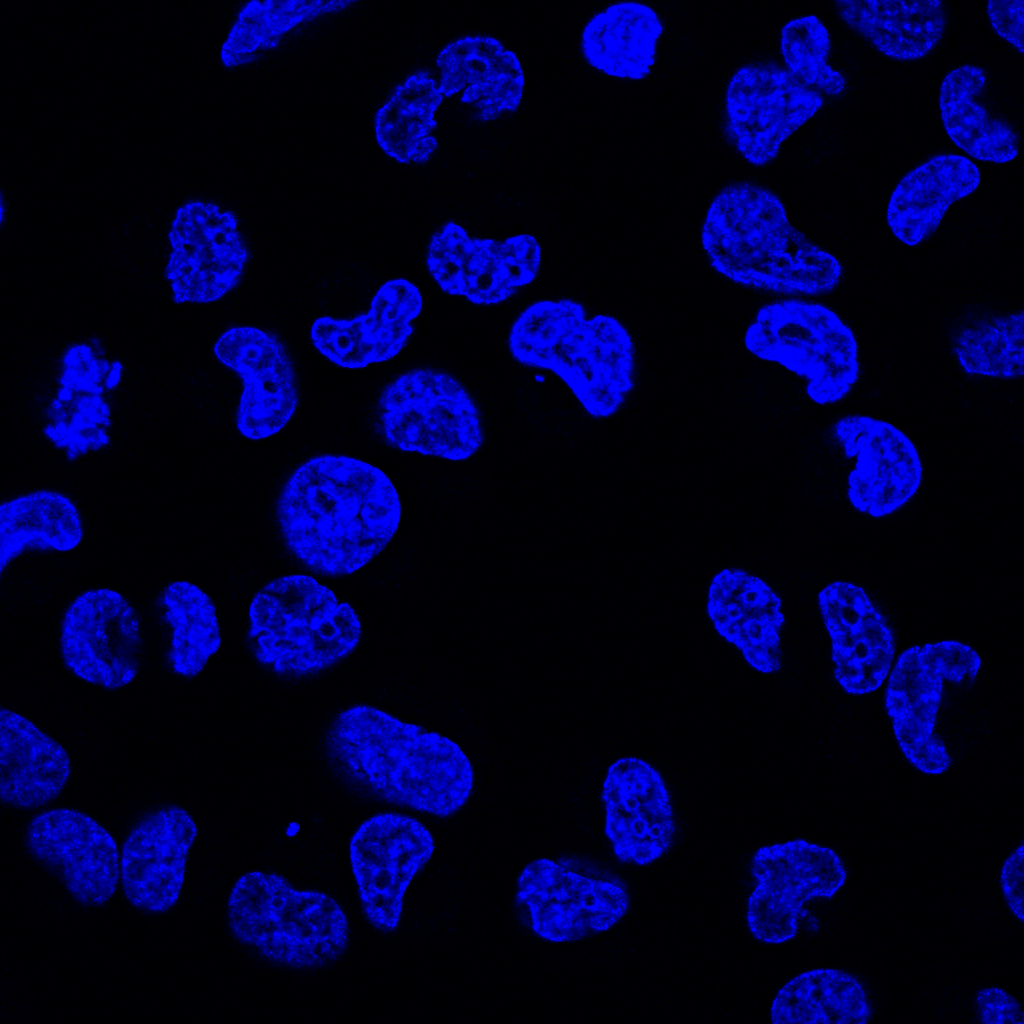

Supplement: Supplemental Information 1 [file peerj-10-14201-s001.zip › Supplemental file Raw data-0824/Supplemental file-Raw data for Figure 5A-1.tif]

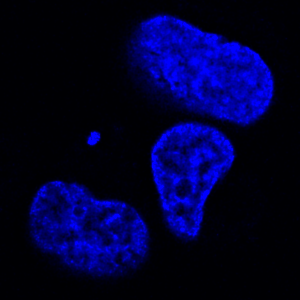

Supplement: Supplemental Information 1 [file peerj-10-14201-s001.zip › Supplemental file Raw data-0824/Supplemental file-Raw data for Figure 5A-2.tif]

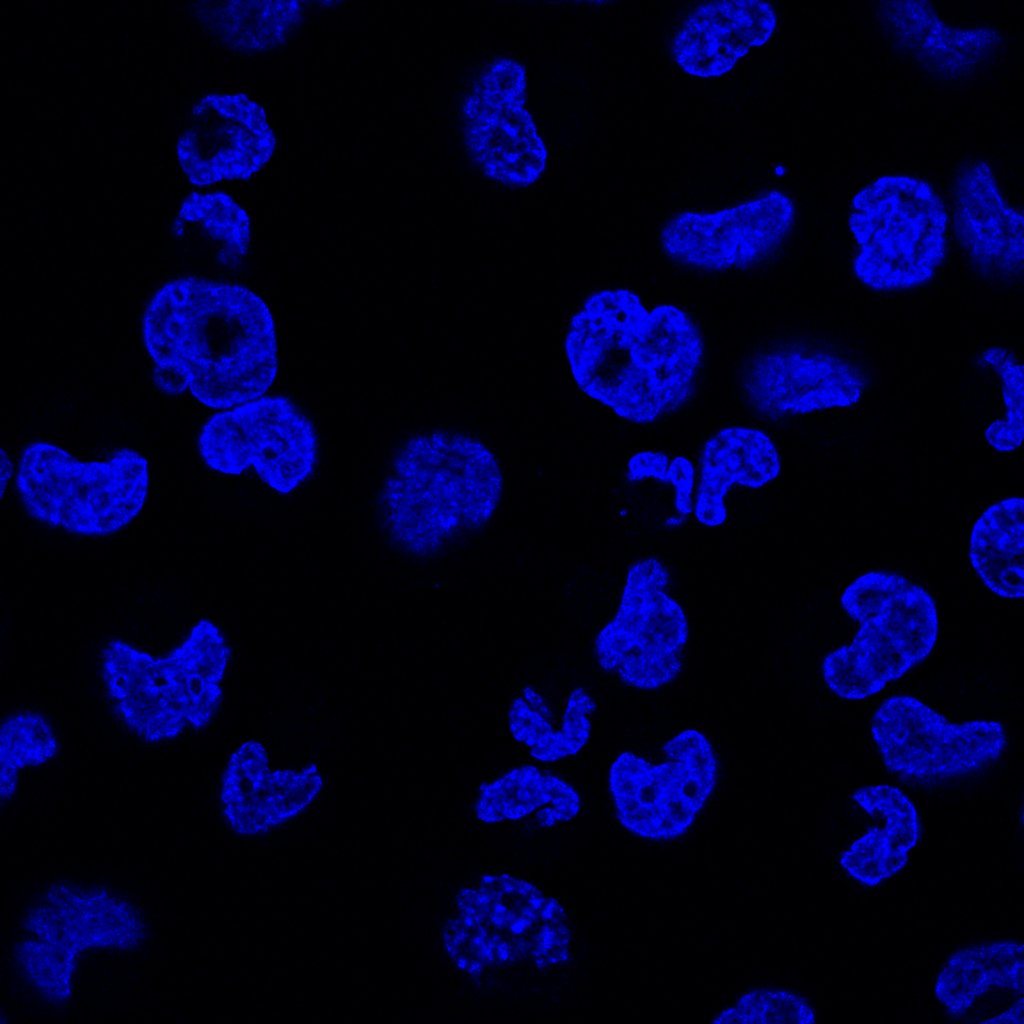

Supplement: Supplemental Information 1 [file peerj-10-14201-s001.zip › Supplemental file Raw data-0824/Supplemental file-Raw data for Figure 5C-1.jpg]

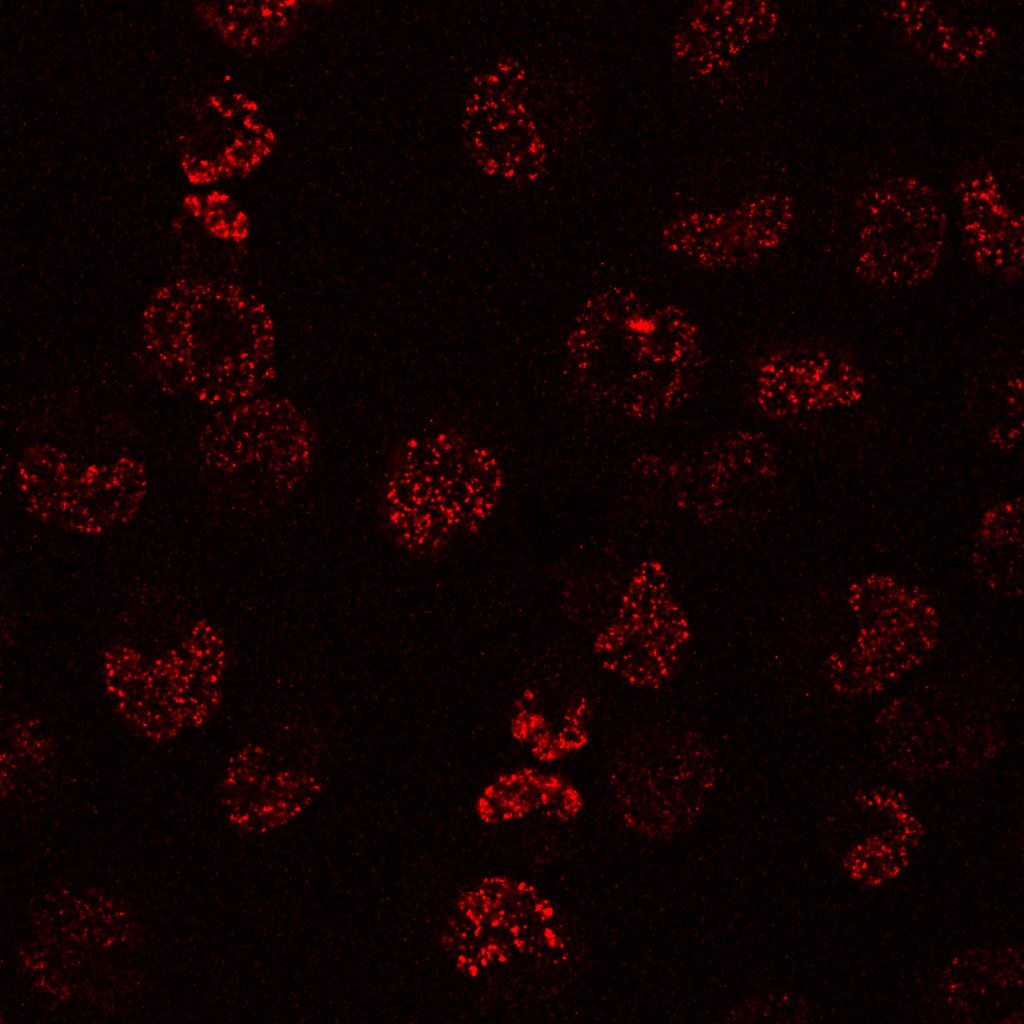

Supplement: Supplemental Information 1 [file peerj-10-14201-s001.zip › Supplemental file Raw data-0824/Supplemental file-Raw data for Figure 5C-2.jpg]

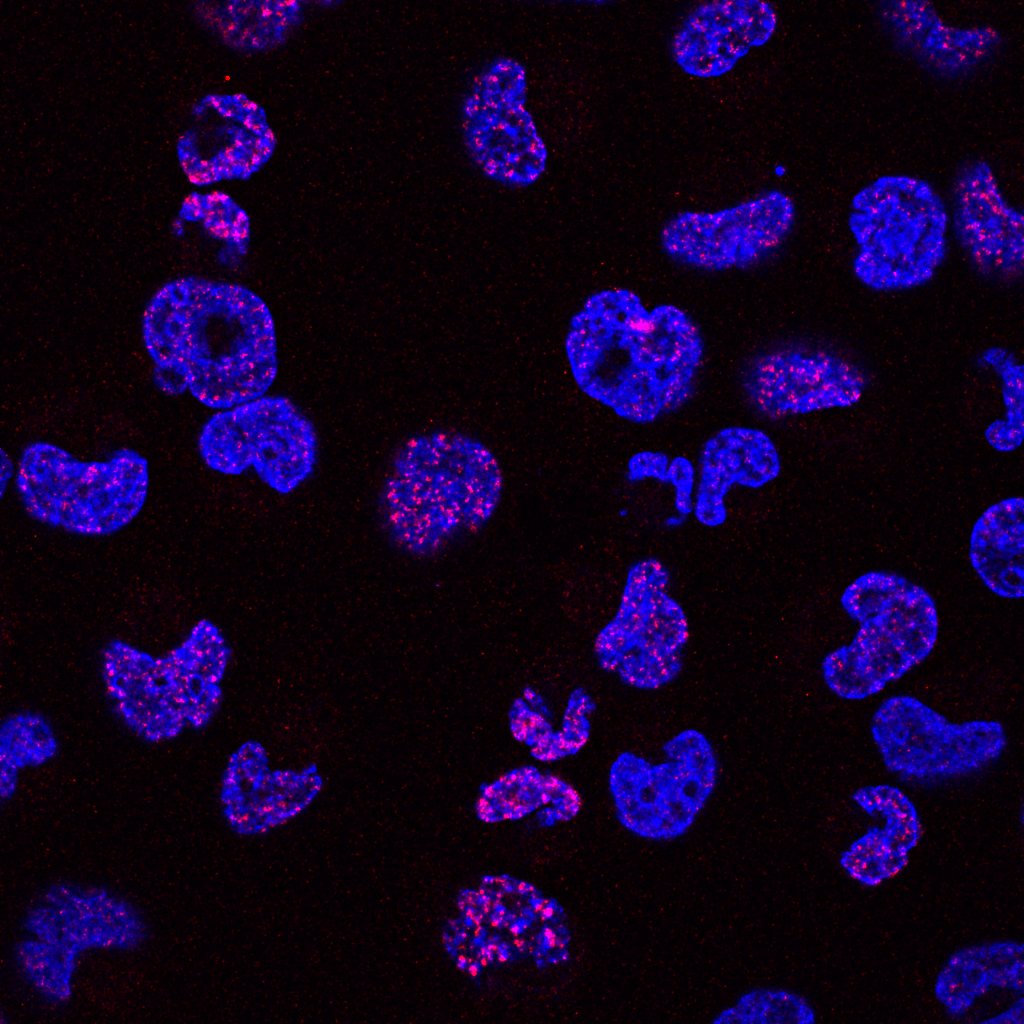

Supplement: Supplemental Information 1 [file peerj-10-14201-s001.zip › Supplemental file Raw data-0824/Supplemental file-Raw data for Figure 5C-3.jpg]

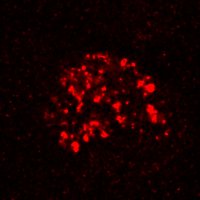

Supplement: Supplemental Information 1 [file peerj-10-14201-s001.zip › Supplemental file Raw data-0824/Supplemental file-Raw data for Figure 5C-4.jpg]

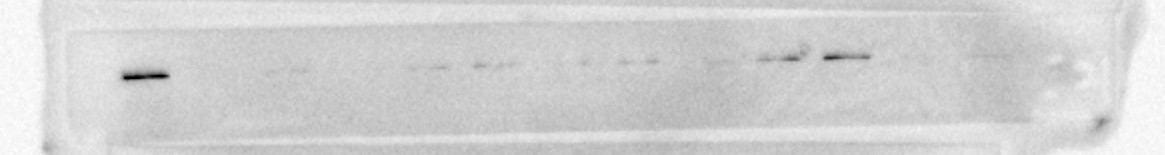

Supplement: Supplemental Information 1 [file peerj-10-14201-s001.zip › Supplemental file Raw data-0824/Supplemental file-Raw data for Figure 5D-1.jpg]

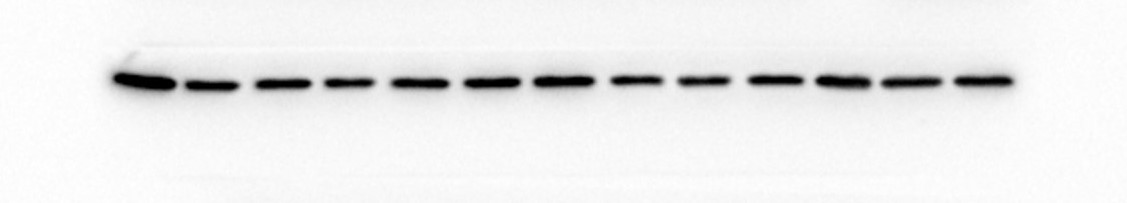

Supplement: Supplemental Information 1 [file peerj-10-14201-s001.zip › Supplemental file Raw data-0824/Supplemental file-Raw data for Figure 5D-2.jpg]

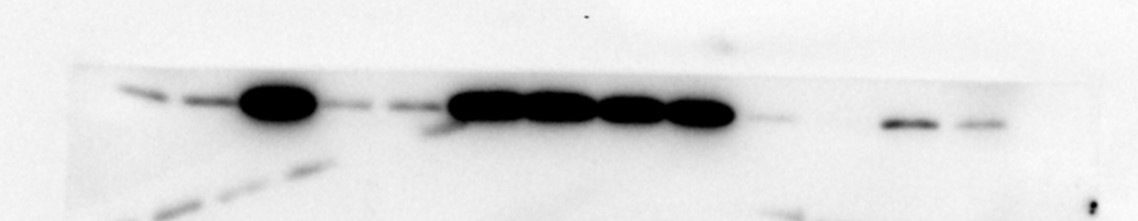

Supplement: Supplemental Information 1 [file peerj-10-14201-s001.zip › Supplemental file Raw data-0824/Supplemental file-Raw data for Figure 5D-3.jpg]

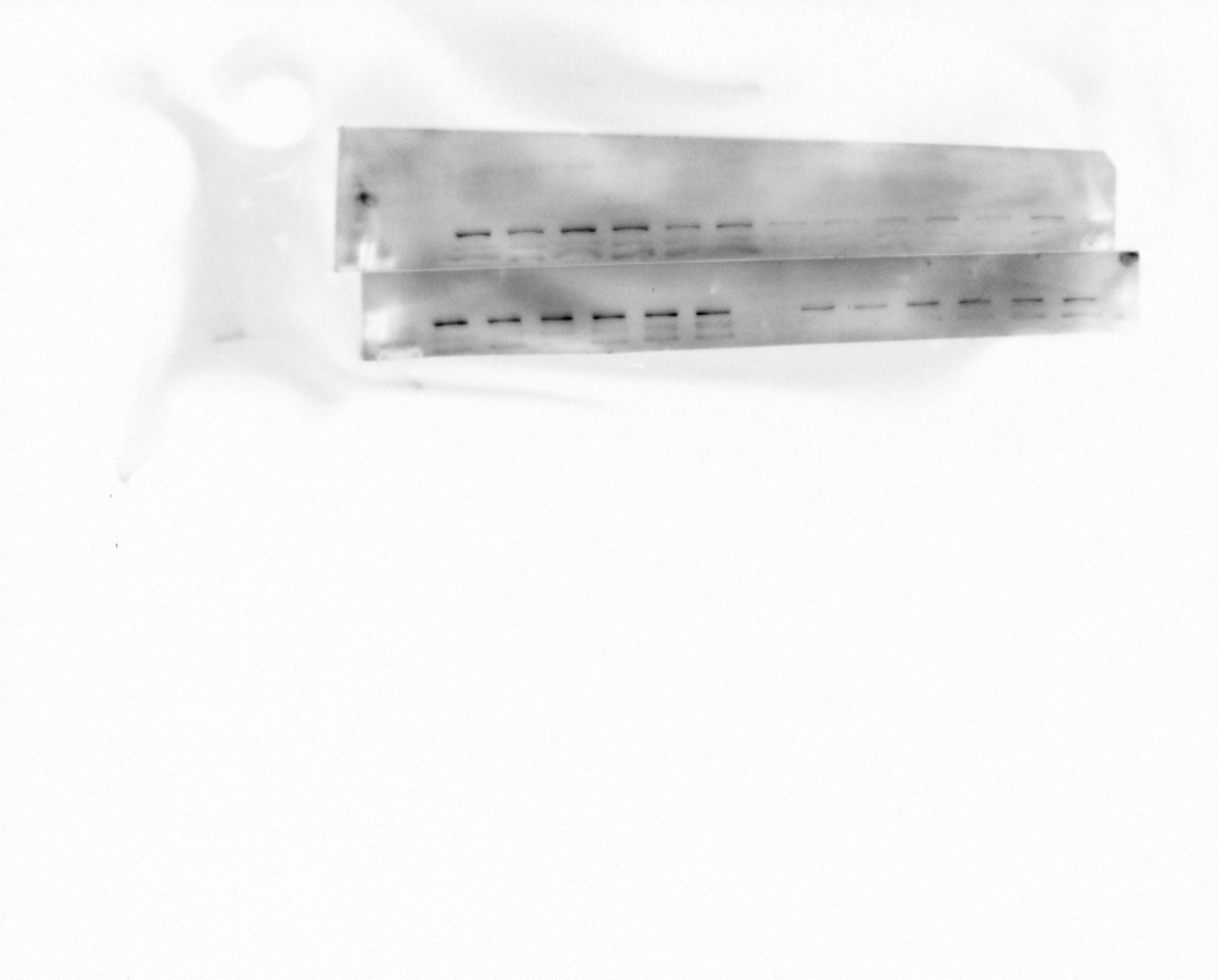

Supplement: Supplemental Information 1 [file peerj-10-14201-s001.zip › Supplemental file Raw data-0824/Supplemental file-Raw data for Figure 5F-1.jpg]

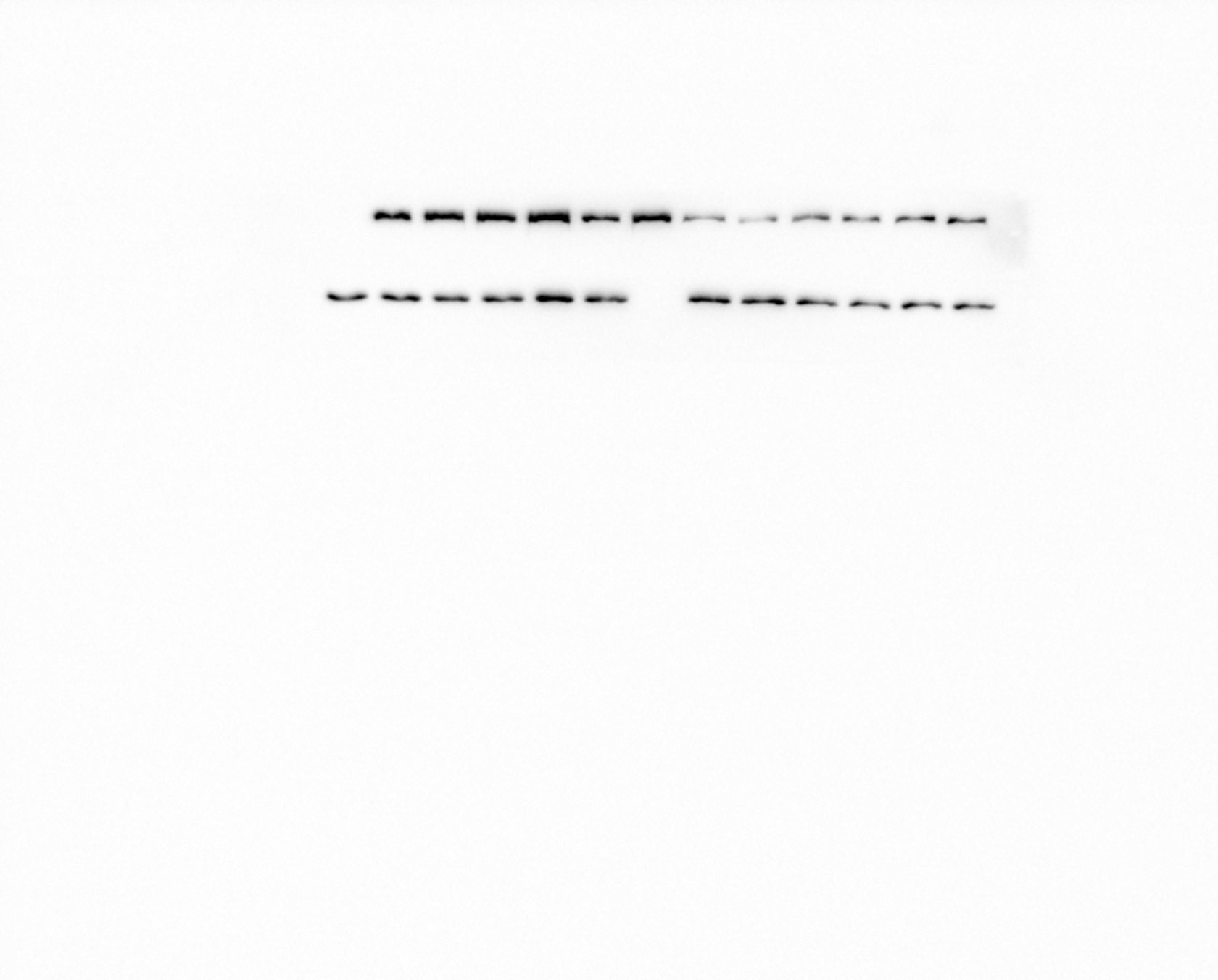

Supplement: Supplemental Information 1 [file peerj-10-14201-s001.zip › Supplemental file Raw data-0824/Supplemental file-Raw data for Figure 5F-2.jpg]

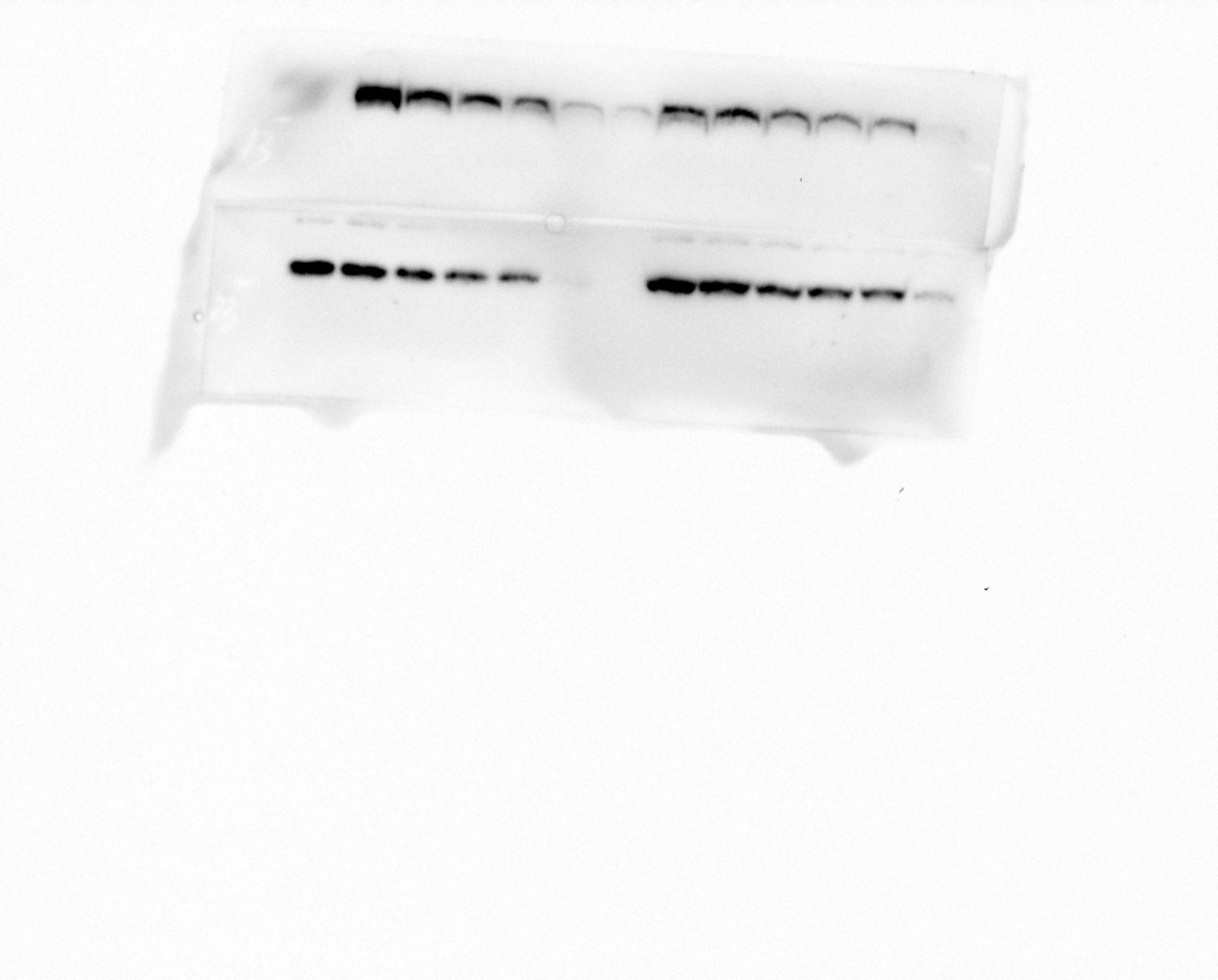

Supplement: Supplemental Information 1 [file peerj-10-14201-s001.zip › Supplemental file Raw data-0824/Supplemental file-Raw data for Figure 5F-3.jpg]
